# Physicochemical Modifications on Thin Films of Poly(Ethylene Terephthalate) and Its Nanocomposite with Expanded Graphite Nanostructured by Ultraviolet and Infrared Femtosecond Laser Irradiation

**DOI:** 10.3390/polym14235243

**Published:** 2022-12-01

**Authors:** René I. Rodríguez-Beltrán, Javier Prada-Rodrigo, Ana Crespo, Tiberio A. Ezquerra, Pablo Moreno, Esther Rebollar

**Affiliations:** 1 Grupo de Aplicaciones del Láser y Fotónica (ALF-USAL), Universidad de Salamanca, Pl. de la Merced s/n, 37008 Salamanca, Spain; 2 CONACYT-Centro de Investigación Científica y de Educación Superior de Ensenada, Unidad Foránea Monterrey, Alianza Centro 504, PIIT, Apodaca 66629, Mexico; 3 Instituto de Química Física Rocasolano, Consejo Superior de Investigaciones Científicas (IQFR-CSIC), Serrano 119, 28006 Madrid, Spain; 4 Instituto de Estructura de la Materia, Consejo Superior de Investigaciones Científicas (IEM-CSIC), Serrano 121, 28006 Madrid, Spain

**Keywords:** laser-induced periodic surface structures, thin polymer films, femtosecond laser nanostructuring, wettability, surface energy, nanomechanical properties

## Abstract

In this work, the formation of laser-induced periodic surface structures (LIPSS) on the surfaces of thin films of poly(ethylene terephthalate) (PET) and PET reinforced with expanded graphite (EG) was studied. Laser irradiation was carried out by ultraviolet (265 nm) and near-infrared (795 nm) femtosecond laser pulses, and LIPSS were formed in both materials. In all cases, LIPSS had a period close to the irradiation wavelength and were formed parallel to the polarization of the laser beam, although, in the case of UV irradiation, differences in the formation range were observed due to the different thermal properties of the neat polymer in comparison to the composite. To monitor the modification of the physicochemical properties of the surfaces after irradiation as a function of the laser wavelength and of the presence of the filler, different techniques were used. Contact angle measurements were carried out using different reference liquids to measure the wettability and the solid surface free energies. The initially hydrophilic surfaces became more hydrophilic after ultraviolet irradiation, while they evolved to become hydrophobic under near-infrared laser irradiation. The values of the surface free energy components showed changes after nanostructuring, mainly in the polar component. Additionally, for UV-irradiated surfaces, adhesion, determined by the colloidal probe technique, increased, while, for NIR irradiation, adhesion decreased. Finally, nanomechanical properties were measured by the PeakForce Quantitative Nanomechanical Mapping method, obtaining maps of elastic modulus, adhesion, and deformation. The results showed an increase in the elastic modulus in the PET/EG, confirming the reinforcing action of the EG in the polymer matrix. Additionally, an increase in the elastic modulus was observed after LIPSS formation.

## 1. Introduction

Polymeric materials are fascinating due to their low cost, ease of manufacture, durability, lightness, and mechanical and chemical characteristics, among other properties. Therefore, they are widely used in scientific and technological fields and in products used in daily life [1,2]. Sometimes, their properties are insufficient or need to be adapted to be used in specific applications. The control of the nanostructure is one way to provide these improvements. In a first approximation, semi-crystalline polymers, which are inherently nanostructured, were obtained from various crystallization processes. This type of material has both crystalline and amorphous regions. While the former provides the polymer with high hardness and thermal stability, the amorphous zones give the material a certain elasticity and impact resistance. The properties strongly depend on the degree of crystallinity, the size and distribution of the crystals, and the properties of the interface between the amorphous and crystalline regions [3].

A different way to modify or improve the properties of polymers is the incorporation of additives into their matrix [4,5]. For instance, the exceptionally high values of the elastic modulus and other mechanical properties of additives open the door to a new generation of polymeric nanocomposites with optimized mechanical properties [6,7]. Other properties also experience a significant improvement compared to their original values, such as improvement in electrical [8] and thermal [4,9] conductivities and impermeability to gases [10]. Expanded graphite (EG), which is a material derived from natural graphite [11], has been used to reinforce different polymers, leading to an improvement in their thermal, mechanical, electrical, and barrier properties [12,13,14].

Additionally, surface processing of polymers has significant importance today. In particular, there is an interest in generating nanostructures with complex morphologies in polymers or polymer-based composites to produce functional surfaces [15,16]. The nanostructures generated in these materials may have interesting wettability properties for applications, such as the prevention of frost formation [17] or water harvesting [18], or adhesion properties that mimic characteristics exhibited by some living beings in nature [19]. The polymer surface and its properties may be modified by irradiation with electron beams [20], ion beams [21], or plasmas [22], inducing changes both in the morphology and the chemistry and related properties such as wettability. Other nanostructuring techniques, such as those based on lithography [23], may be used to nanostructure polymer surfaces, but they have some limitations in addition to lacking versatility and simplicity in comparison to other techniques, such as laser processing [24]. One of these laser-based techniques is the formation of laser-induced periodic surface structures (LIPSS) [25,26]. In the particular case of polymers, LIPSS have been observed with pulse durations in the nanosecond [27,28], picosecond [29,30], and femtosecond range [31,32] and at wavelengths ranging from the ultraviolet to the infrared [29,32]. While, in the case of irradiation with ns laser pulses, it is necessary that the polymer absorbs efficiently at the laser wavelength, this is not relevant for irradiation with shorter pulses. Specifically, the formation of LIPSS on polymeric surfaces using fs pulses has been carried out with UV and NIR irradiation. Although the material absorbs efficiently under UV wavelengths, it is also possible to form LIPSS using fs pulses at wavelengths for which the material has low linear absorption coefficient values. In this case, due to the high intensities that are reached by fs laser irradiation, the absorption on the outer layer of the polymer comes about by nonlinear mechanisms, such as multiphoton absorption and ionization processes. The results are periodic structures parallel to the polarization of the laser beam with periodicities coinciding nearly to the wavelength and an absence of an ablation scenario.

It has been reported that the presence of LIPSS involves a modification of the properties at the surface, leading, for instance, to the induction of important changes in their optical, mechanical, and tribological properties and wettability, biocompatibility, etc. [24,33,34]. In particular, control of the wettability of surfaces has become a very interesting topic to study because of its importance in many applications. It is increasingly employed in applications for anti-icing [35], self-cleaning [36], and mimicking the functionalities of the skin of animals on technical materials [37].

Previously, we reported on the formation of LIPSS on the surface of poly(ethylene terephthalate) (PET) and the nanocomposite of PET and EG using UV nanosecond laser pulses [38], and the physicochemical modifications induced in the materials were assessed. The results showed an increase in the hydrophilicity of the surfaces after laser irradiation, with an increase in the surface free energy and, in particular, of its polar component. In the present work, we present a systematic study of the LIPSS formation on thin films of PET and PET/EG using femtosecond laser pulses with two different wavelengths in the ultraviolet and IR regions. On the one hand, the possibility of tuning and controlling the size of the periodic structures is demonstrated, and, on the other, the effect on the physicochemical properties as a function of the laser wavelength and of the filler presence is studied. In particular, we study the modifications induced by laser irradiation by considering the morphology using atomic force microscopy (AFM), the wettability and surface energy by measuring the contact angle (CA) with different liquids, and the adhesion and mechanical properties using a colloid probe and quantitative nanomechanical microscopy techniques.

## 2. Materials and Methods

### 2.1. Materials

Thin films of PET and PET reinforced with EG (0.4 wt.% EG) were prepared. The materials were produced by the in situ polymerization method reported by Paszkiewicz et al. [39]. EG (SGL Carbon SE, Bonn, Germany), with an average thickness of expanded agglomerates of 450–560 nm and graphene platelets from 16 μm to 46 μm (99%) in size, was added to the reaction mixture, but, first, it was combined with ethandiol in order to split agglomerates and to improve further exfoliation. Some of the relevant properties of the PET and PET/EG composites are listed in Table S1 [40]. 

The spin coating technique was used to cover silicon substrates (100) (Wafer World Inc., Florida, USA). Prior to the deposition, the substrates were cleaned in an ultrasonic bath with acetone and isopropanol. Both polymer and composite were dissolved in trifluoroacetic acid (Sigma-Aldrich^®^, Darmstadt, Germany, reagent ≥ 98 %) using a concentration of 15 g/L for 3 h at room temperature. In the preparation of the thin films, ~0.2 mL of the polymer solution was dropped onto the substrate using a pipette. Immediately, samples were spun for 2 min at 2400 rpm. The thickness and average roughness (Ra) of thin films were determined by atomic force microscopy (AFM), and the results are listed in Table 1.

### 2.2. Laser Irradiation

Laser irradiation was performed with the fundamental (795 nm, 120 fs FWHM) and the 3rd harmonic (265 nm, 265 fs FWHM) of a femtosecond laser system, which consisted of a Ti:Sa oscillator (Tsunami, Spectra Physics^®^, Mountain View, CA, USA) and a regenerative amplifier (Spitfire, Spectra Physics^®^). At a repetition rate of 1 kHz, the linearly polarized pulses were focused on the samples by using an achromatic doublet (f = 100 mm) for the fundamental and a plano-convex lens (f = 100 mm) for the UV laser pulses. Samples were placed on a motorized XYZ translation stage. A photograph of the experimental set-up can be seen in Figure S1.

The formation of the structures was studied by varying the irradiation time (number of pulses) and the laser fluence. The number of pulses was controlled by using an electromechanical shutter and neutral filters; a λ/2 waveplate and a linear polarizer were used to adjust the laser energy. In addition, a thermopile detector (407 A, Spectra Physics^®^) was utilized to measure the average power of the beam. Experiments were performed in the air at room temperature and ambient humidity.

### 2.3. Characterization

We used atomic force microscopy in tapping mode to characterize the surface topography. An AFM Multimode 8 (Bruker^®^, Karlsruhe, Germany) system with the controller Nanoscope V (Bruker^®^) and the software Nanoscope Analysis 1.50 (Bruker^®^) was selected for these measurements. The tips were silicon NSG30 (NT-MDT^®^, Moscow, Russia) with a curvature radius of ~6 nm, a nominal resonant frequency of 320 kHz, and a typical spring constant of 40 N/m.

We determined the wettability and surface energy of the materials, before and after irradiation, by measuring their contact angle (CA) with both apolar and polar liquids. The sessile drop technique was used, employing a pocket goniometer PG2 (FIBRO system^®^, Stockholm, Sweden) at room temperature and ambient humidity for water, glycerol, and paraffin oil. We carried out eight measurements for every different sample–liquid pair for non-irradiated samples and samples irradiated with 1000 pulses at 13.8 mJ/cm^2^ with fs UV pulses and 10,000 pulses and 87 mJ/cm^2^ under fs NIR irradiation. 

The samples were analyzed by Raman spectroscopy using a Renishaw Raman InVia Reflex Spectrophotometer (Renishaw Iberica S.A.U., Gavá, Spain) equipped with a Leica Microscope and an electrically refrigerated CCD camera. An excitation of 532 nm and a 50× magnification objective were employed. Acquisition time and accumulations were 10 s and 10, respectively, and the spectral resolution was 2 cm^−1^. The diameter of the laser spot on the sample was diffraction limited by the objective lens and calculated to be ca. 1 μm. 

The colloidal probe technique was used to characterize adhesion strength at the micro scale in samples before and after laser irradiation. Measurements were carried out with a colloidal tip CPFM_SiO_2_–A/Au (NT-MDT^®^) with a sphere radius between 5 and 9 μm. Three sets of curves per sample were recorded. The cantilever spring constant was measured by the thermal tune method and found to be around 1.6 N/m.

The nanomechanical properties of the films were evaluated simultaneously to the topography by the PeakForce Quantitative Nanomechanical Mapping (PF-QNM) method using the same AFM equipment and RTESPA300 probes (Bruker^®^). The cantilever spring constant was calculated by the Sader method [41] and found to be around 22–27 N/m. Tip radius calibration was performed before every measurement against a polystyrene standard of known elastic modulus (2.7 GPa) and found to be in the range of 7–12 nm.

## 3. Results

### 3.1. LIPSS Formation

We irradiated the different materials with fs laser pulses in the UV range at 265 nm and in the NIR range at a wavelength of 795 nm. Irradiation with two different wavelengths in different materials can result in specific features in the morphology and properties of induced structures. Potentially, it offers the possibility of generating nanostructures using not only irradiation at an excitation wavelength at which these materials absorb efficiently but also at a wavelength where the linear absorption of these materials is almost zero (see Figure S2). 

**Figure 1:**
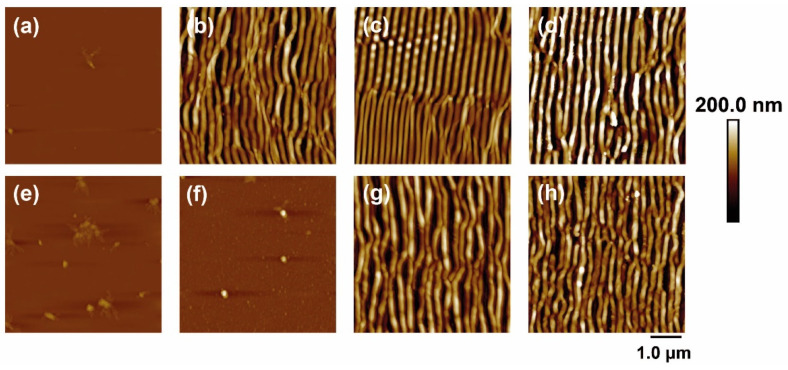
AFM topography images of PET (**top row**) and PET/EG 0.4 wt.% (**bottom row**) thin films: (**a**,**e**) before and after irradiation with (**b**,**f**) 11.6 mJ/cm^2^, (**c**,**g**) 13.8 mJ/cm^2^, and (**d**,**h**) 15.7 mJ/cm^2^. Laser irradiation was performed with 5000 pulses at a wavelength of 265 nm.

**Figure 2:**
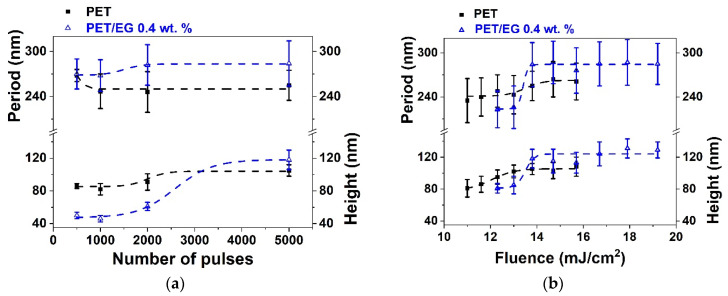
Heights and periods of LIPSS induced on thin films of PET (■) and PET/EG 0.4 wt.% (Δ) as a function of: (**a**) the number of pulses at a constant fluence of 13.8 mJ/cm^2^ and (**b**) the fluence at a constant number of pulses of 5000. Laser irradiation was performed at a wavelength of 265 nm. Dotted lines are displayed as visual guides. The data shown correspond to the average and standard deviation of 50 measurements.

Figure 1 shows some AFM images of PET and PET/EG before and after irradiation with fs UV laser pulses at different laser fluences with a constant number of pulses of 5000. The dependence of the period and height of the LIPSS with the fluence were obtained as a function of the fluence and the number of pulses and are plotted in Figure 2.

It can be seen that it was possible to induce the formation of LIPSS parallel to the polarization vector of the incident beam in both materials, with periods close to the irradiation wavelength. LIPSS were formed from 500 to 5000 pulses in the range of fluences of 11.0–15.7 mJ/cm^2^ in the case of PET and 12.3–19.2 mJ/cm^2^ in the case of PET/EG. The range of formation of LIPSS as a function of the number of pulses was the same for both the pure polymer and the nanocomposite (Figure 2a). Likewise, the values of period and height followed a similar trend. They increased gradually as the number of pulses increased, reaching a constant value for both magnitudes from 2000 to 3000 pulses. Maximum values close to irradiation wavelengths were obtained for the period of the structures in both materials, although slightly larger values were observed for the nanocomposite. The final values for height were close to 100 nm, but, for irradiation with a low number of pulses, the height of the LIPSS was significantly smaller for PET/EG in comparison to PET. There was also a difference in the range of fluences in which LIPSS were generated. In particular, the range for PET/EG surfaces was wider than in the case of PET (Figure 2b). The values of period and height gradually increased until they reached a maximum value of around 14 mJ/cm^2^. This can be explained by considering, on the one hand, the higher crystallinity of PET/EG samples (Table S1) and, on the other, the different thermal properties caused by the presence of the carbon nanoadditive. The increase in thermal conductivity and heat capacity in polymer/carbon composites relative to the neat polymer has already been reported in the literature [42,43]. Thus, the surface temperature reached upon irradiation is smaller in the case of the nanocomposite. A lower surface temperature leads to a higher superficial viscosity, and, thus, the material rearrangement, and LIPSS formation, is more difficult. Furthermore, a higher thermal conductivity induces faster cooling of the material, and the feedback effect involved in LIPSS formation is less efficient [44].

**Figure 3:**
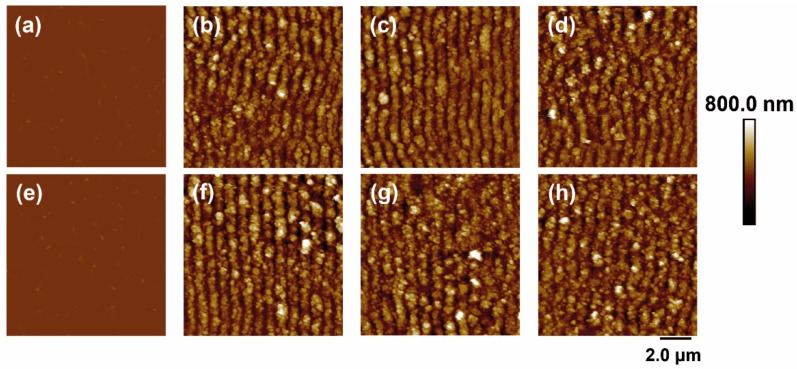
AFM topography images of PET (**top row**) and PET/EG 0.4 wt.% (**bottom row**): (**a**,**e**) before and after irradiation with a fluence of (**b**,**f**) 76 mJ/cm^2^, (**c**,**g**) 87 mJ/cm^2^, and (**d**,**h**) 94 mJ/cm^2^. Laser irradiation was performed with 50,000 pulses at a wavelength of 795 nm.

**Figure 4:**
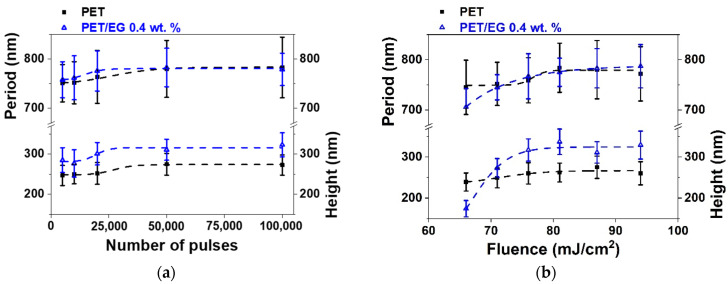
Heights and periods of LIPSS induced on thin-film sample surfaces supported by PET silicon (■) and PET/EG 0.4 wt.% (Δ) as a function of: (**a**) the number of pulses at a constant fluence of 87 mJ/cm^2^; and (**b**) fluence at a constant pulse number of 50,000. Laser irradiation was performed at a wavelength of 795 nm. Dotted lines are displayed as visual guides. The data shown correspond to the average and standard deviation of 50 measurements.

In the case of irradiation with laser pulses of fs in the NIR range (795 nm), the results are shown in Figure 3. For both materials, again, LIPSS were formed parallel to the polarization vector of the incident beam and with periods close to the irradiation wavelength. The values of height and period of the structures as a function of fluence and number of pulses are shown in Figure 4.

The range of formation of LIPSS as a function of the number of pulses was the same for both materials, following the same trend in period and height values (Figure 4a). They increased gradually as the number of pulses increased, reaching a constant value for both magnitudes from 50,000 pulses. Maximum values close to 775 nm were obtained for the period in both materials. The final values for height were close to 275 nm and 325 nm for the raw polymer and the composite, respectively, i.e., slightly higher for the case where EG is present. 

Regarding the dependence on fluence (Figure 4b), the formation range was the same for the two materials, taking place from 66 to 94 mJ/cm^2^. The values in period and height followed similar trends in both cases, gradually increasing as the pulse fluence increased until they reached a constant maximum value of around 81 mJ/cm^2^. The nanostructures for PET and the nanocomposite reached a maximum period of around 780 nm. The height of the structures was close to 275 nm and 325 nm for PET and PET/EG, respectively.

The formation of nanostructures was observed for irradiation at both wavelengths. The results show the possibility of controlling the morphology and size of structures through the laser parameters, such as wavelength, fluence, and the number of pulses. Irradiation with fs UV pulses leads to the formation of nanostructures due to mechanisms analogous to those described for ns UV pulses, where thermal effects play a relevant role [38]. The materials studied have a high linear absorption coefficient at 265 nm [38]. However, it was also possible to nanostructure these materials by irradiating them with pulses of fs NIR, a wavelength at which the linear absorption was negligible. Therefore, in this second case, the absorption of laser radiation by materials took place by nonlinear mechanisms. As previously reported, due to the longer penetration depths and/or multiphoton ionization processes, IR fs laser processing tends to produce high-aspect-ratio features [45].

Samples nanostructured with LIPSS were further characterized from the point of view of the physicochemical and nanomechanical properties. For that purpose, samples were irradiated at 265 nm using a laser fluence of 13.8 mJ/cm^2^ and 1000 pulses and at 795 nm using a fluence of 87 mJ/cm^2^ and 10,000 pulses. 

### 3.2. Determination of Contact Angles for Different Test Liquids

**Figure 5:**
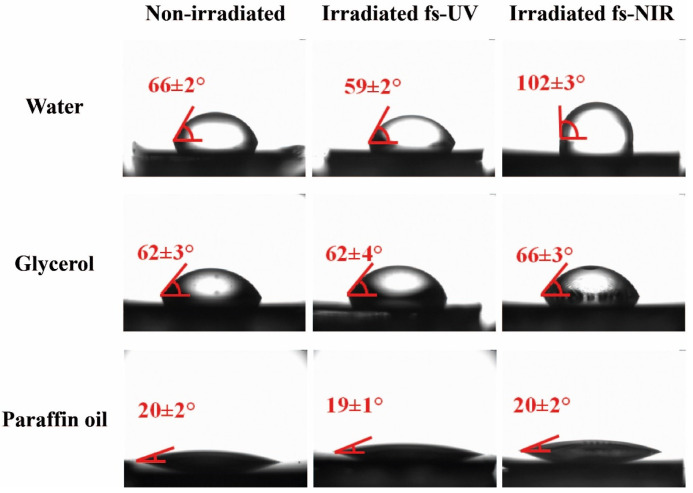
Images of droplets from three different test liquids on unirradiated and irradiated surfaces with fs UV and fs NIR pulses of PET/EG 0.4 wt.%. In all images, the values of the corresponding contact angle between the drop of the test liquid and the surface are indicated.

The sessile drop technique was used to measure the contact angles (CA) of the laser-treated surfaces using water, glycerol, and paraffin oil. These test liquids were chosen due to their polarity characteristics (Table S2), which were necessary for carrying out the subsequent calculation of the components of the surface free energy. For instance, Figure 5 shows the results for PET/EG, and the value of each contact angle, shown in red in each image, was the result of the statistical average of three individual measurements. The contact angles for both materials are listed in Table 2.

Before being irradiated, the surfaces did not present any difference in the values of the contact angles for the different liquids between the neat polymer and its respective nanocomposite, as previously reported for similar samples [38]. Although higher values were expected for the water contact angle due to the hydrophobic character of the EG additive, the wettability was similar to that exhibited by the surface of the neat matrix. Even if the additive was well dispersed in the polymeric matrix, its concentration in the polymer was negligible. Therefore, its presence was insufficient to induce a significant effect on the wettability of the surfaces.

After irradiation, it can be stated that, for UV irradiation, the materials increased their hydrophilicity. For the other liquids, no significant changes were observed. On the one hand, this increase in hydrophilicity can be explained through the Wenzel model [46], which describes a homogeneous wetting regime and predicts a change in surface wettability due to an increase in surface roughness. Specifically, in this case, there was a solid with an initial hydrophilic character that became more hydrophilic because of the nanostructured process. On the other hand, the formation of LIPSS is a superficial phenomenon, and chemical modifications may take place in the outermost layer of the material (a few tens of nanometers) [47]. 

In the case of NIR irradiation, it was observed that the water contact angle increased significantly in both materials, indicating that the surfaces became hydrophobic after laser structuring. In principle, considering the larger size of the obtained structures, both in period and in depth, the Cassie–Baxter model can be used to explain the hydrophobic character of these samples after laser irradiation. Due to the increase in surface roughness, the liquid did not penetrate the cavities, allowing the generation of vapor pockets between such roughness. This model considers that the contact angle can be increased even when the contact angle of the liquid on the unmodified surface is less than 90°. Since the structures induced with fs NIR pulses had dimensions (height and period) more prominent than those induced by pulses of fs UV, the liquid did not penetrate or partially penetrate between the cavities of the LIPSS, giving rise to the hydrophobic character of the samples. However, further inspection of the irradiated surfaces regarding possible chemical modifications was needed, and it is shown in the following section.

It is important to mention that, for PET/EG, as observed for the non-irradiated samples, the irradiated ones did not present significant differences in the contact angle measured using the different liquids independently of the irradiation wavelength. This indicates that the additive was also well dispersed in the polymer matrix after irradiation.

### 3.3. Surface Free Energy

By using the CA reported above, we calculated the surface energy and its components. In the present study, we used the model by Owens, Wendt, Rabel, and Kaelble (work model) [48]. This model uses the Young’s equation to determine the surface total energy $\gamma_{S}^{\mathrm{TOT}}$ and the dispersive $\gamma_{S}^{d}$ and polar $\gamma_{S}^{p}$ components. The surface free energies of the components of the liquid probes are listed in Table S2, and the calculated values are summarized in Table 3.

**Figure 6:**
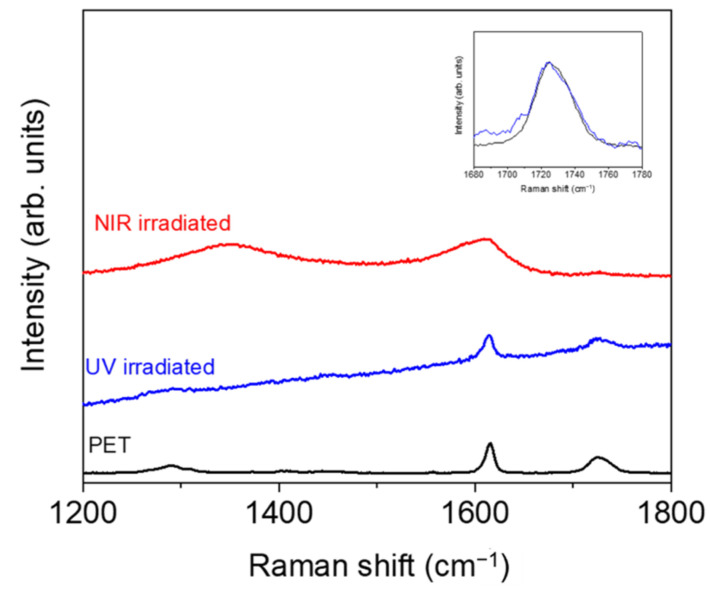
Micro-Raman spectra (λ_exc_ = 532 nm) of non-irradiated PET and PET irradiated with UV and NIR wavelengths. Spectra were shifted vertically for the sake of comparison. The inset shows magnification of the region around 1720 cm^−1^ for PET and PET irradiated with UV pulses (in this case, spectra were base-line corrected and normalized).

Before irradiation, there were no significant differences between the neat polymer and its nanocomposite since no significant differences were observed in the contact angles with the different liquids, and, after irradiation, both PET and PET/EG behaved in the same way. After UV irradiation, the dispersive component did not change significantly, while the polar component increased, and the total surface energy increased slightly. The increase in the polarity of samples may be related to photooxidation on the surface upon UV irradiation, as reported previously for different polymers irradiated with both lasers [47,49] and lamps [50]. Specifically, the increase in the polar component for UV was reported to be a consequence of the formation of polar hydrophilic species, such as carbonyl, hydroxyl, and carboxyl groups, by a reaction with oxygen from the air, as has been previously reported [51]. To obtain some information about the possible chemical modifications, we analyzed the irradiated samples by Raman spectroscopy, and the results for PET are shown in Figure 6. We focused on the region from 1200 to 1800 cm^−1^, where the bands corresponding to C(O)–O stretching (1290 cm^−1^), ring mode 8a (1615 cm^−1^), and the stretching C=O vibration (1726 cm^−1^) were observed. The spectrum of the PET irradiated with UV light displayed an increase in the fluorescence background with respect to the non-irradiated sample, indicating that additional modifications occurred upon irradiation. Additionally, the band at 1726 cm^−1^ was wider after irradiation, indicating a larger contribution of C=O type groups, in agreement with the increase in polarity obtained from the CA measurements. Similar results were obtained for PET/EG, as displayed in Figure S3.

Under fs NIR pulsed irradiation, the dispersive component increased, and the polar component and the total surface energy decreased. The behavior of PET and the composite remained the same. The changes observed, particularly significant in the case of the polar component, were related to the formation of new functional groups at the polymer surface. In the case of irradiation with NIR pulses, previous studies by Raman spectroscopy [51] showed carbonization after nanostructuring. Thus, it can be inferred that the changes in polarity after structuring were caused by a change in the chemistry of the material in its outermost layer where LIPSS were present. The Raman spectrum in this case showed the appearance of new bands at around 1340 and 1580 cm^−1^, which were assigned to amorphous carbon [52]. This corroborates that carbonization of the film surface takes place upon irradiation and explains the change from hydrophilic to hydrophobic surfaces. The same results were obtained for PET/EG (Figure S3).

### 3.4. Modification of Adhesion Force

**Figure 7:**
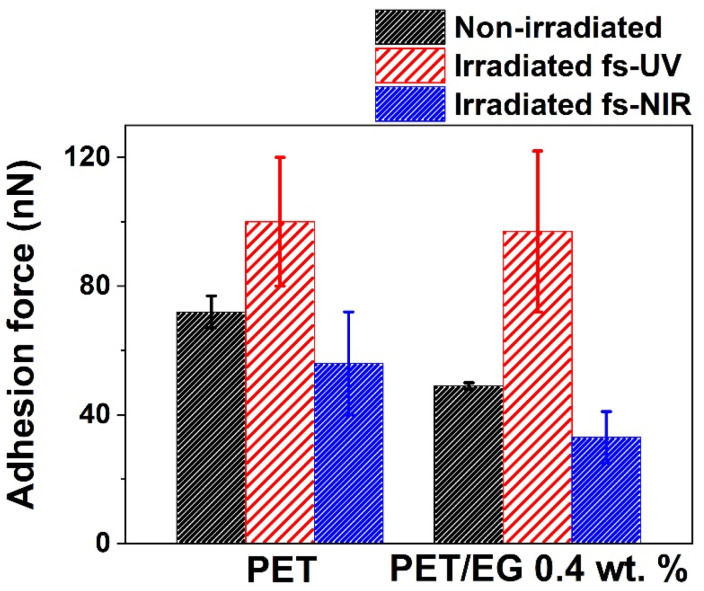
Adhesion force determined by the colloidal tip technique. The data shown correspond to the average and standard deviation of three measurements.

Force–distance measurements were carried out using the AFM and a colloidal tip. Figure 7 presents the results obtained with the colloidal tip technique on the surfaces. Regarding the samples before irradiation, the adhesion force of the polymer was similar but slightly higher than that of the composite. When these surfaces were irradiated with fs pulses in the UV range, both materials increased their adhesion force compared to the non-irradiated surface. In contrast, for NIR irradiation, the adhesion force decreased. In both cases, the values of the composite in comparison to the neat polymer were similar but slightly smaller. 

As in the case of CA measurements, the colloidal tip technique also gave information about the changes produced in the outermost layer of the solid studied. The results showed two different trends since, in one case, the magnitude of this property increased (UV pulses, as was also observed for ns laser irradiation [38]), while, in the other, it decreased (NIR pulses). These differences followed the same tendencies obtained in the case of CA results, i.e., there was an increase in the adhesion force and the surface free energy for the UV irradiation case and a decrement of both magnitudes in the case of NIR irradiation. The decrease in the adhesion force has been previously studied on micro- and nanostructured surfaces and reported in the literature [53,54]. In these studies, it was concluded that the decrease in the adhesion properties of the surfaces after being structured was due to the reduction of the contact area of the tip with the sample. However, in the case of UV irradiation, this decrease seemed to be compensated by the enhanced adhesion expected from the higher surface polarity. So, in that case, nanostructuring was accompanied by an increase in hydrophilicity, surface energy, and surface adhesion, which may be relevant for the development of different applications.

### 3.5. Nanomechanical Properties

**Figure 8:**
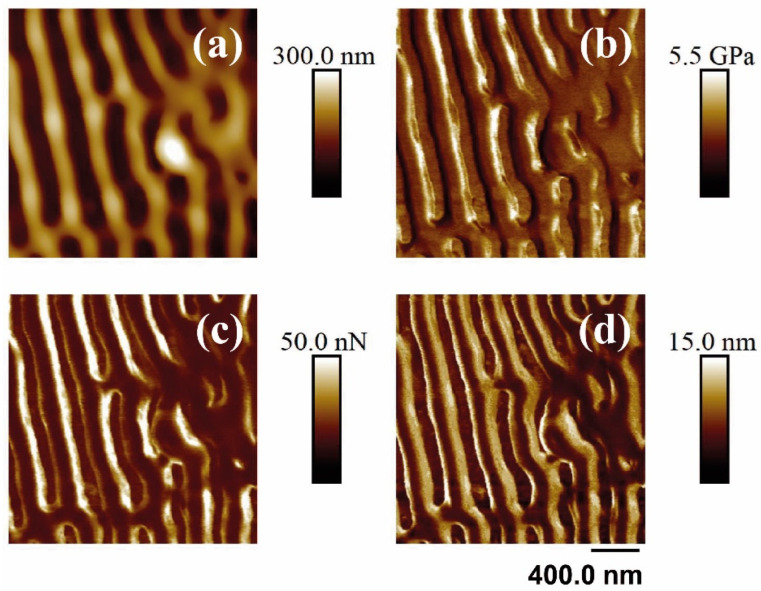
PF-QNM-AFM (2 × 2 μm^2^) mapping of the topography (**a**), Young’s module (**b**), adhesion force (**c**), and deformation (**d**) corresponding to the surface supported in unirradiated and irradiated PET silicon. The irradiation parameters were 5000 pulses, 13.8 mJ/cm^2^, 265 nm.

**Figure 9:**
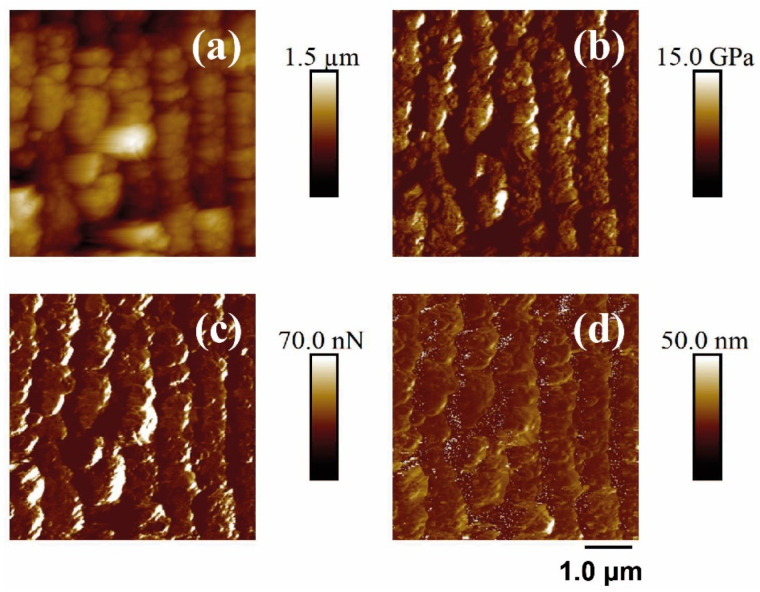
PF-QNM-AFM (5 × 5 μm^2^) mapping of the topography (**a**), Young’s modulus (**b**), adhesion force (**c**), and deformation (**d**) corresponding to the surface supported in unirradiated and irradiated PET silicon. The irradiation parameters were 10,000 pulses, 87 mJ/cm^2^, 795 nm.

Finally, the PF-QNM-AFM technique was performed to measure the nanomechanical properties of the samples before and after laser treatment. Figure 8 and Figure 9 show the different properties measured on a PET surface irradiated with fs UV and fs NIR irradiation, respectively. Table 4 summarizes the measurement results. 

As can be observed in Table 4, for PET and PET/EG, a clear improvement in the elastic modulus was seen when the polymer matrix was reinforced with the additive EG. Paszkiewicz et al. [55] determined, by tensile testing, the value of the elastic modulus in PET and PET/EG compounds. This study demonstrated an apparent increase in the elastic modulus as the concentration of the additive increases. The trend started with a value of 2.08 ± 0.14 GPa for the raw polymer and reached a maximum value of 2.67 ± 0.10 GPa for the polymer matrix with a maximum concentration of additive (0.4 wt.%).

Upon irradiation with fs UV pulses, the value of the elastic modulus increased for both materials, and the elastic modulus of PET was still smaller than that of the nanocomposite. The adhesion force presented a different trend since the value for PET decreased after LIPSS formation while, for PET/EG, the adhesive properties remained almost unaltered. Finally, the deformation value for PET increased in the presence of LIPSS.

Under NIR irradiation, elastic modulus also increased, while the values of the adhesion force for PET and its compound did not exhibit any change after irradiation. Regarding deformation, it increased for PET after irradiation, while, for PET/EG, it remained constant. 

The increase in the values of the elastic modulus observed for the irradiated samples with the two different wavelengths may be explained by considering confinement of the material along a direction. The value of the elastic modulus for PET of 2.6 GPa has been reported for injection-molded PET [56]. Changes in mechanical properties were observed due to a confinement effect in one or two dimensions of the material [57,58]. In the case of LIPSS, the impact of laser nanostructure was such that it increased the local mechanical stability of the material, contributing to an increase in its elastic properties. This direct implication in relation to the robustness of the material in the areas affected by laser irradiation is due to the fact that confinement provides better dissipation of mechanical stress, such as that produced by the tip of AFM when indenting the material [58].

Information reported in the literature about measurements in adhesion strength with PF-QNM-AFM in these materials is very scarce [59,60]. By comparing these works and the present study, it was deduced that the values measured by PF-QNM-AFM were lower than those obtained by the colloidal tip technique. For flat samples, some research has shown that the adhesion force is proportional to the radius of the tip [61]. Its value increases with the size of the AFM tip because the actual contact area is also larger [53]. This happens because the Derjaguin, Muller, and Toporov (DMT) model determines the adhesion between particles and a substrate, assuming that all materials are elastically deformed, and interactions occur both in the contact zone and outside it, predicting a linear dependence of the adhesion force with the radius of the particle [62].

The PF-QNM-AFM technique offers a better approach as it is able to measure at a point (on a nanoscale). The mapping of large areas can be obtained, simultaneously measuring topography and other nanomechanical properties such as elastic modulus and deformation.

After nanostructuring, the value of the adhesion force changes slightly. As mentioned previously, laser irradiation may induce the formation of new functional groups at the surface, affecting the physicochemical properties of the irradiated material. Therefore, the measurement of the adhesion force, even using the PF-QNM-AFM technique, remains connected with the physicochemical properties in the material as it happens when measuring adhesion with a colloidal tip. It should also be taken into account that the tip–surface interaction continues to be governed by forces of attraction or repulsion such as van der Waals, electrostatic, magnetic, and capillary forces [63]. When a colloidal tip is used, its larger size allows for more tip–surface interactions and, in fact, enhances others that otherwise would have almost negligible magnitudes [64]. With PF-QNM-AFM, the interaction with the material remains the same, but the contact area is much smaller due to the size of the tip.

## 4. Conclusions

LIPSS parallel to the polarization vector of the incident laser beam and with a period close to the laser wavelength were formed by irradiation both with fs UV and NIR pulses on thin films of PET and its composite PET/EG 0.4 wt.% supported on silicon substrates. 

While the original surfaces had a hydrophilic character before irradiation, upon laser irradiation in the UV, samples became more hydrophilic, which can be explained through Wenzel’s model. In addition, an increase in the polar components of the free surface energy was observed, suggesting the formation of polar hydrophilic species. In contrast, upon irradiation with fs NIR, the surfaces acquired a hydrophobic state. Using the Cassie–Baxter model, it is possible to explain this behavior. In addition, the changes in the values of the polar components suggest surface chemical changes that also influence the change in the wettability of surfaces.

The magnitude of the adhesion force, obtained with the colloidal tip technique, increased after laser irradiation with UV pulses, while, for NIR irradiation, it decreased. The decrease in the adhesion can be, in principle, explained by the reduction of the contact area of the tip with the sample. On the other hand, the increase in the adhesive properties of structured samples may be related to the interfacial interaction between the tip and the surfaces.

Using the PF-QNM-AFM technique, the reinforcing action of the EG in the polymer matrix was confirmed. Additionally, an increase in the elastic modulus was observed after LIPSS formation, which must be attributed to the phase changes experienced by the materials and the rearrangement and confinement they undergo during the laser irradiation. 

In conclusion, it is possible to modify PET and PET/EG surfaces, also modifying the wetting, surface energy, and adhesion properties in a different and controllable way depending on the laser wavelength used. This has important implications for the use and performance of PET and its nanocomposites.

## Figures and Tables

**Table 1:** Thickness and average roughness of PET and PET/EG 0.4 wt.% thin films as a result of three different measurements.

Material	Thickness (nm)	Ra (nm)
PET	195 ± 14	0.6 ± 0.2
PET/EG 0.4 wt.%	178 ± 10	1.7 ± 0.1

**Table 2:** Contact angles of the different probe liquids (water, glycerol, and paraffin-oil) of non-irradiated (NI) and irradiated (fs UV and fs NIR pulses) on PET and PET/EG 0.4 wt.% surfaces, obtained by the sessile drop technique. The data listed correspond to the average and standard deviation of three different measurements.

Material	Surface Condition	Water (°)	Glycerol (°)	Paraffin Oil (°)
PET	NI	69 ± 2	59 ± 2	19 ± 3
	fs-UV	62 ± 5	64 ± 3	20 ± 2
	fs-NIR	107 ± 5	63 ± 2	17 ± 3
PET/EG 0.4 wt.%	NI	66 ± 2	62 ± 3	20 ± 2
	fs-UV	59 ± 2	62 ± 4	19 ± 1
	fs-NIR	102 ± 3	66 ± 3	20 ± 2

**Table 3:** Surface energy and its components (mJ/m^2^) calculated by using the OWRK method of non-irradiated (NI) and irradiated (fs UV and fs NIR pulses) on PET and PET/EG 0.4 wt.% surfaces obtained from the average data listed in Table 2. The errors were estimated at ca. 10%.

Surface Energy Components (mJ/m^2^)				
Material	Surface Condition	$\gamma_{S}^{d}$	$\gamma_{S}^{p}$	$\gamma_{S}^{\mathrm{TOT}}$
PET	NI	26.8	12.1	38.9
	fs-UV	24.2	15.8	40.0
	fs-NIR	34.9	0.2	35.1
PET/EG 0.4 wt.%	NI	25.4	13.7	39.1
	fs-UV	24.3	17.5	41.8
	fs-NIR	32.5	0.7	33.2

**Table 4:** Elastic modulus, adhesion force, and deformation of non-irradiated (NI) and irradiated (fs UV and fs NIR pulses) on PET and PET/EG 0.4 wt.% surfaces, obtained by PF-QNM-AFM technique. The data listed correspond to the average and standard deviation of three different measurements.

Material	Surface Condition	Elastic Modulus (GPA)	Adhesion Force (nN)	Deformation (nm)
PET	NI	1.5 ± 0.1	7.9 ± 0.5	2.4 ± 0.2
	fs-UV	2.4 ± 0.1	4.8 ± 0.6	6.1 ± 0.3
	fs-NIR	2.8 ± 0.3	7.1 ± 1.2	3.6 ± 0.6
PET/EG 0.4 wt.%	NI	5.1 ± 0.2	7.4 ± 0.5	2.0 ± 0.1
	fs-UV	5.6 ± 0.1	8.6 ± 0.6	2.1 ± 0.2
	fs-NIR	4.2 ± 0.6	7.7 ± 0.8	2.1 ± 0.3

## Data Availability

Data available on request from the corresponding author.

## Supplementary Materials

The following supporting information can be downloaded at: https://www.mdpi.com/article/10.3390/polym14235243/s1, Figure S1: Experimental set-up used for LIPSS formation with NIR fs irradiation; Figure S2: Absorption spectra corresponding to PET and PET/EG 0.4 wt.%; Figure S3: Micro-Raman spectra (λexc = 532 nm) of non-irradiated PET/EG and PET/EG irradiated with UV and NIR wavelengths. Spectra were shifted vertically for the sake of comparison; Table S1: Weight average molar mass (Mw), number average molar mass (Mn), dispersity (Mw/Mn), and mass crystallinity (Xc) for the PET and its composite; Table S2: Surface energy and its components (mJ/m^2^) of the probe liquids.
