# Supplementary material for: Physicochemical Modifications on Thin Films of Poly(Ethylene Terephthalate) and Its Nanocomposite with Expanded Graphite Nanostructured by Ultraviolet and Infrared Femtosecond Laser Irradiation
Source: Polymers (Basel). 2022 Dec 1;14(23):5243. doi: 10.3390/polym14235243 (PMC9737047; doi:10.3390/polym14235243)
Supplement: Supplementary file 1 [file polymers-14-05243-s001.zip › polymers-2029569-supplementary.pdf]

Supplementary Information

Physicochemical modifications on thin films of polyethylene-terephthalate and its nanocomposite with expanded graphite nanostructured by ultraviolet and infrared femtosecond laser irradiation

René I. Rodríguez-Beltrán[1,2*], Javier Prada-Rodrigo[1,3], Ana Crespo[4], Tiberio A. Ezquerra[4], Pablo Moreno[1], Esther Rebollar[3*]

[1] Grupo de Aplicaciones del Láser y Fotónica (ALF-USAL), Universidad de Salamanca, Pl. de la Merced s/n, 37008 Salamanca, Spain

[2] CONACYT- Centro de Investigación Científica y de Educación Superior de Ensenada, Unidad Foránea Monterrey, Alianza Centro 504, PIIT, Apodaca, Nuevo León CP 66629, México

[3] Instituto de Química Física Rocasolano, Consejo Superior de Investigaciones Científicas (IQFR-CSIC), Serrano 119, 28006 Madrid, Spain

[4] Instituto de Estructura de la Materia, Consejo Superior de Investigaciones Científicas (IEM-CSIC), Serrano 121, 28006 Madrid, Spain

**Corresponding authors: rrodrigu@cicese.mx, e.rebollar@csic.es.*

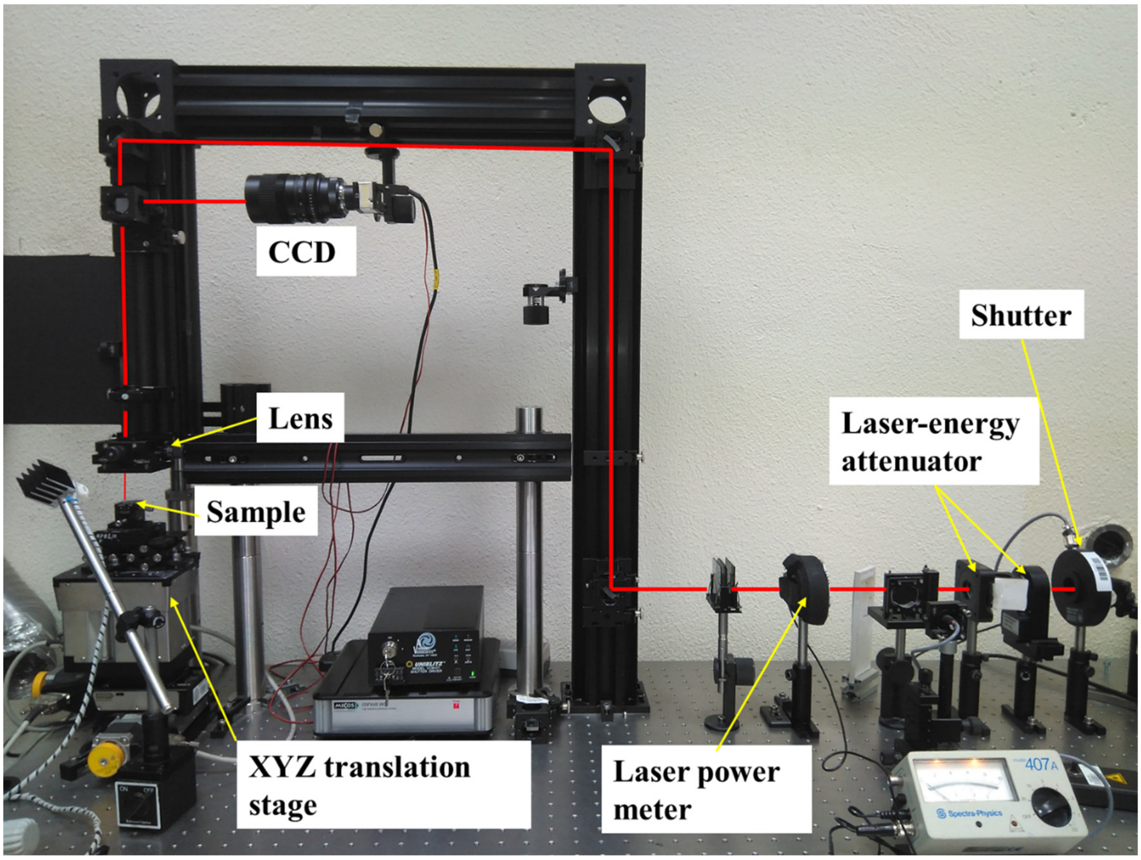

Figure S1. Experimental set-up used for LIPSS formation with NIR fs irradiation.

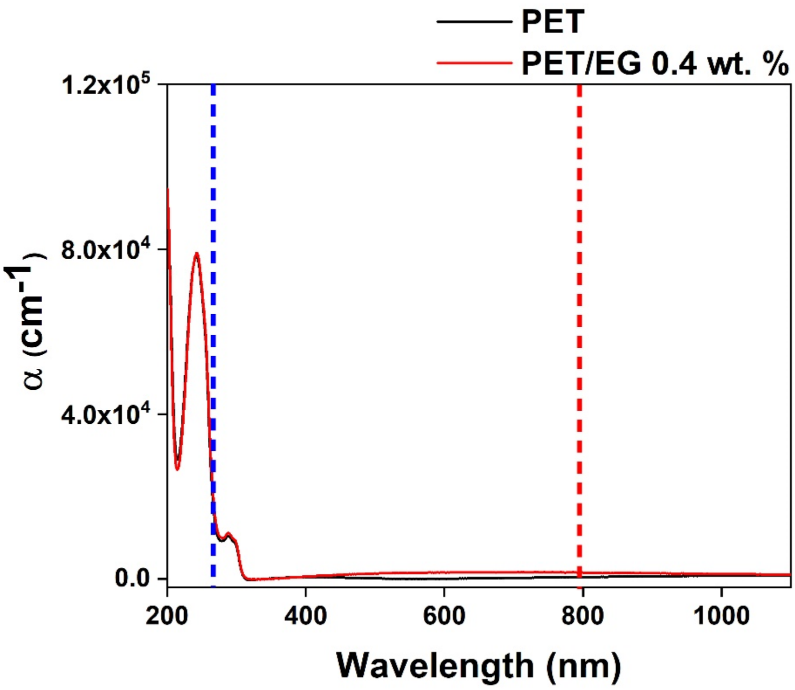

Figure S2. Absorption spectra corresponding to PET and PET/EG 0.4 wt.%

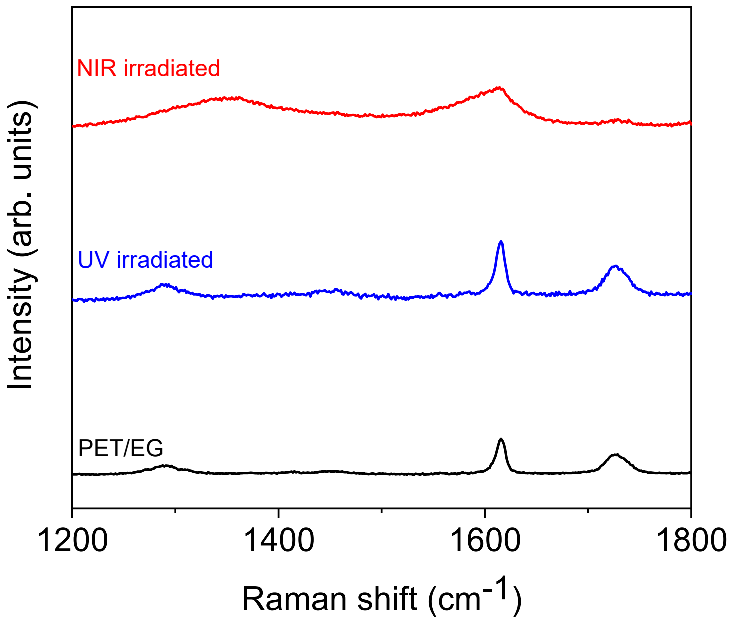

Figure S3. Micro-Raman spectra (λ_{exc} = 532 nm) of non-irradiated PET/EG and PET/EG irradiated with UV and NIR wavelengths. Spectra were shifted vertically for the sake of comparison.

Table S1. Weight average molar mass (Mw), Number average molar mass (Mn), dispersity (Mw/Mn) and mass crystallinity (Xc) for the PET and its composite [39]

	M_w x10^4 g/mol	M_n x10^4 g/mol	M_w/M_n	X_c (%)
PET	4.69	1.95	2.41	12
PET/EG 0.4 wt. %	3.85	1.56	2.47	34.7

Table S2. Surface energy and its components (mJ/m^2) of the probe liquids [40].

	**Surface energy components (mJ/m^2)**				
**Material**	γ_S^d	γ_S^p	γ_S^-	γ_S^+	γ_S^{TOT}
**Water**	21.8	51.0	25.5	25.5	72.8
**Glycerol**	34.0	30.0	57.4	3.92	64.0
**Paraffin-oil**	28.9	0	0	0	28.9
